# Formulation and Characterization of Orally Dissolving Thin Films containing the German cockroach *Blatella germanica* (Bla g 2) Allergen

**Published:** 2014

**Authors:** Qingqing Chen, Russell Martin, Stephen W. Hoag, Robert A. Wood, Hai-Quan Mao, Corinne Keet

**Affiliations:** 1 Department of Materials Science and Engineering, Whiting School of Engineering, Johns Hopkins University, 3400 North Charles Street, Baltimore, MD 21218; 2 School of Pharmacy, University of Maryland at Baltimore, 20 North Pine Street, Baltimore, MD 21201; 3 Department of Pediatrics, Johns Hopkins School of Medicine, 600 North Wolfe Street, Baltimore, MD 21287; 4 Translational Tissue Engineering Center, School of Medicine, Johns Hopkins University, 400 North Broadway, Baltimore, MD 21231; 5 Whitaker Biomedical Engineering Institute, School of Medicine, Johns Hopkins University, 720 Rutland Avenue, Baltimore, MD 21205

**Keywords:** Cockroach, immunotherapy, *Bla g 2*, thin film, drug delivery, ELISA

## Abstract

Allergy and asthma are among the most common chronic diseases of childhood. Cockroach allergy is an important contributor to asthma morbidity, with a prevalence of 17 to 41%. Immunotherapy has been shown to be an effective treatment for other allergies that contribute to asthma, but several factors have limited its use for cockroach allergy. In this work, a sublingual immunotherapy (SLIT) formulation of orally dissolving thin film has been developed for the treatment of hypersensitivity to the German cockroach Bla g 2 allergen. The formulation allows for the incorporation of up to 25 μg/film of the allergen protein, and the film's mucoadhesiveness prolongs the effect of the allergen with the potential for enhanced efficacy. The potency and dose uniformity of the SLIT formulation were characterized by enzyme-linked immunosorbent assay (ELISA), and other physicochemical properties were evaluated by spectroscopic or mechanistic methods. The films were uniform in weight and thickness, and demonstrated substantial physical strength to allow easy manipulation during manufacturing and dosing. The dosage uniformity, in vitro disintegration and in vitro dissolution profiles of the films were within the acceptance criteria in the United States Pharmacopeia. The developed SLIT methodology possesses the potential to significantly improve immunotherapy for both food and inhalant allergies in adults and children.

## INTRODUCTION

Inner city residents suffer a disproportionate asthma prevalence and morbidity compared to their suburban counterparts in the United States. Although many factors may be responsible for this disparity, there is a growing body of evidence to show that the indoor environment plays a key role in asthma related health issues in inner-city populations [1]. At least 50% of inner-city homes have been shown to have clinically relevant levels of cockroach allergen [2, 3], and 30% of suburban homes show detectable levels of the allergen [4, 5]. In the National Cooperative Inner City Asthma Studies, 37% of inner-city children were sensitized to cockroach, and those that were both sensitized and exposed to high levels of cockroach had more than 3 times as many hospitalizations compared to non-sensitized and/or non-exposed children [3]. Numerous studies subsequent to this pioneering work have confirmed that the combination of cockroach allergy and cockroach exposure is one of the most important factors leading to high morbidity in inner-city children with asthma [6].

Subcutaneous immunotherapy (SCIT) for treatment of allergies associated with asthma is a well-established approach in general [7]. However, safety concerns, poor acceptability of injections and the necessity for many visits over years currently limit the utility of SCIT for the treatment of asthma in the inner-city [8]. Sublingual immunotherapy may offer improved safety and acceptability [9]. However, in a recent pilot study conducted by the NIH-sponsored Inner-City Asthma Consortium, using liquid cockroach allergen extracts, cockroach SLIT elicited far lower immunological changes than in studies using grass SLIT [10]. One possible reason for the poor response is the difficulty in achieving a dose likely to be effective for SLIT with liquid extracts; in the Inner-City Asthma Consortium study, the maximum daily dose was 16.8 μg of Bla g 2, and this was only achieved with twice daily dosing. Other concerns with liquid extracts include the possibility of aspiration, particularly in young children who may have difficulty holding an extract under the tongue for several minutes [10]. Therefore in this current study, we developed a SLIT formulation against the German cockroach Bla g 2 allergen using oral dissolving thin films as a viable alternative to both SCIT and liquid extract treatments.

Film drug delivery is a method of delivering drugs using a thin film that dissolves when in contact with liquid media (e.g. saliva), and thus delivers the active compound locally. This methodology allows for safer and more convenient therapy; the film's muco-adhesiveness also prolongs the effect of the allergen with the potential for enhanced efficacy [11, 12]. The film effectively stabilizes the immunogenicity of the allergenic protein, and delivers a higher, clinically relevant dose. The films are formulated with up to 25 μg of Bla g 2 allergen per dose. We characterized the film formulation based on appearance, physicochemical properties, allergen content using enzyme-linked immunosorbent assay (ELISA*),* film disintegration and Bla g 2 dissolution from the film. We also investigated the influence of water content on the stability of the filmstrips.

## MATERIALS AND METHODS

### Materials

Defatted ground German cockroach source material was purchased from Greer Inc. (Lenoir, North Carolina) and kept frozen until use. Hypermellose (hydroxypropyl methylcellulose) viscosity grade Methocel® E 15 LV Premium was obtained from The DOW Chemical Company (Midland, Michigan). Glycerin NF grade was obtained from Spectrum Chemical MFG Corp. Chocolate flavor was purchased from Abelei Flavors (North Aurora, Illinois) and used as is. Distilled and deionized water used in the formulation and all experimental analyses was obtained through a Millipore Milli Q water purification system. All of the ingredients above were analytical or food grade.

The ELISA kit for Bla g 2 was purchased from Indoor Biotechnologies (Charlottesville, Virginia). The secondary antibody, anti-rabbit HRP (horseradish peroxidase) was obtained from Thermo Scientific. 2,2'-Azino-bis(3-ethylbenzothiazoline-6-sulphonic acid) (ABTS) was purchased from Invitrogen (Grand Island, New York) and reconstituted prior to use.

### Preparation of *Blatella germanica* crude extract

The allergen extract was prepared by adding the powdered crude extract at 20% w/v in deionized water and incubating at 4°C overnight. Afterwards it was centrifuged at 3,000× g for 15 minutes, and the supernatant was collected for use.

### Film Formulation

A solvent casting method was used to prepare the thin films used to deliver the cockroach allergen extract. Solutions of Methocel^®^ prepared at a concentration of 10% w/v (in deionized water) was used to incorporate the Bla g 2 allergen solution. The optimized composition of the film is tabulated in **Table 1**. The allergen extract, Methocel®, glycerin, water, and flavoring were mixed in a suitable vial followed by brief, pulsed ultrasonic defoaming to remove the air bubbles in the solution. The solution was cast onto a flat Teflon® plate with a 5 cm (L) × 2.5 cm (W) × 0.25 cm (D) indentation, which was custom-made. The films were dried overnight in a 40°C oven to evaporate the excess water, then gently peeled off the cast and cut into 1 cm × 2 cm strips. The Bla g 2 content in each filmstrip was assayed using ELISA.

### Weight and Thickness

The weight and thickness of the 1 cm × 2 cm film-strips were measured. Thickness was measured using calibrated digital Vernier Calipers (Cen-Tech). The thickness of each strip was evaluated at five different locations (four corners and one center) [15]. This experiment analyzes the physical uniformity of the films as they are directly related to the uniformity of the allergen dose distribution in the filmstrip.

### Foldability

Foldability was assessed by folding each 1 cm × 2 cm film-strip repeatedly down the lateral midline until breakage occurs or visible cracks begin to appear. A total of three strips were tested. The number of times the strip could be folded without breaking or cracking was recorded as its foldability value.

### Tensile Strength

Tensile strength was measured using a Dynamic Mechanical Analysis instrument (DMA Q800, New Castle, Delaware). The instrument was equipped with one fixed clamp and one movable clamp, which extended to induce strain on the specimen. Displacement was applied length-wise at a constant rate of 100 μm per minute to a maximum extension of 2 mm (10% total elongation). The results were recorded on an engineering stress *vs.* strain curve. The highest point of the curve was reported as the ultimate tensile strength.

### Water Content

To assess the water content of the thin film, the filmstrips were weighed at the end of the casting process, and inserted into separate borosilicate scintillation vials and lyophilized for periods of 2 hours. At the end of each period, the films were extracted and allowed to return to ambient conditions in a desiccator before re-weighing. Afterwards they were placed back into lyophilization. This process was continued until two consecutive weighings did not differ by more than 0.50 mg. The difference in initial and end weight was recorded as the absolute water content of the strip, according to the following formula:

$$\%\;Water\;Content\;=\;\frac{W_{i}\;-\;W_{f}}{W_{i}}\;\times \;100$$

Where *W_i_* is the initial weight of the film, *W_f_* is the final weight of the film after drying.

### Surface pH

The surfaces of the films were wetted slightly with deionized water and its pH measured by bringing a micro-pH electrode (Mettler Toledo InLab Micro electrode) into contact with the wetted surface.

### *In vitro* disintegration test

Disintegration is the physical process by which the film dissolves into a solution. Disintegration time was measured for three strips according to a method previously described by Bala *et al* [9]. Briefly, each filmstrip was dipped in 25 mL of deionized water in a suitable container. Water was gently agitated by a 1-inch Teflon^®^-coated stir bar at 75 rpm. Disintegration time was recorded when the film starts to break apart or disintegrates.

### *In vitro* dissolution test

*In vitro* dissolution study was conducted in accordance with the conditions in United States Pharmacopeia (USP) method <711>, apparatus II. The dissolution medium was 900 mL of freshly deionized water, the temperature was maintained at 37 ± 0.5°C and stirred 75 rpm. At 10, 15, 20, 25, 30, and 45 minutes, 1 mL aliquots of solution samples were extracted and measured on an UV/Vis spectrophotometer (NanoDrop 2000c, Thermo Scientific) at wavelength of 300 nm. The resulting absorbance values were correlated with those of a serially-diluted cockroach protein standard.

### Content Uniformity

The dosage uniformity of the Bla g 2 allergen was quantitated using ELISA assay for ten filmstrips following the guidelines set out by the USP method <905>. Each filmstrip was fully dissolved in 1 mL of deionized water and pre-diluted 100 fold before loaded onto a 96-well microtiter plate for assay. The amount of Bla g 2 was quantitated based on the logarithmic fit of the ELISA standard curve. The Bla g 2 content of each individual filmstrip must be between 85% and 115% of the target dose (25 μg/filmstrip) and the relative standard deviation must be less than or equal to 6.0%. The acceptance value (AV) was calculated according to the following equation:

AV=∣M−X∣+k⋅s

Where *M* is the label claim (use 100%), *X* is the average % of the individual samples compared to label claim, *k* is the acceptability (use 2.4 for n = 10), and *s* is the standard deviation of the sample set. *AV* must be less than 15%, according to the United States Pharmacopeia.

### Stability

Accelerated stability tests were conducted in at 40°C - 75% RH (Relative Humidity) over 2 weeks. To assess the influence of excess water content on the stability of the filmstrips, the strips were placed into three groups with varying degrees of drying. Group 1 was dried under ambient conditions and contained 5% excess water. Group 2 was dried overnight in a 40°C oven and contained 3% of excess water. The drying method for group 2 resembles the conditions employed during the scale-up manufacturing process. Group 3 was subjected to lyophilization procedures identical to the water content analysis described previously; therefore, contained less than 0.1% excess water in the formulation. The samples were sealed individually in containers to simulate the conditions of the final product packaging. The amount of Bla g 2 in the filmstrips was assayed by ELISA on days 3, 7 and 14. The sample preparations for ELISA analysis for stability were identical to the method described above for content uniformity.

## RESULTS AND DISCUSSION

### Film Formulation

**Figure 1:**
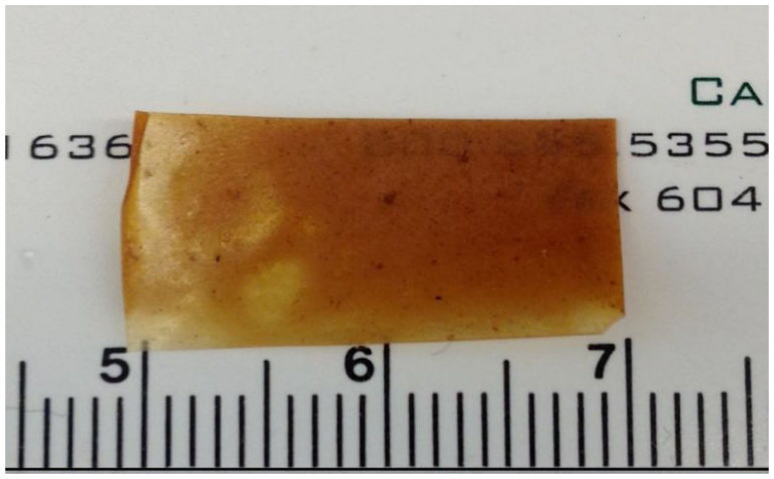
The appearance and dimensions of the oral dissolving thin film strip with 25 μg of Bla g 2 allergen loading.

The optimized formulation incorporated a maximum of 25 μg of Bla g 2 allergen in each 1 cm × 2 cm filmstrip, as determined by ELISA assay. The selected composition demonstrated the most satisfactory physical properties. The films dried tack-free, could be cleanly peeled from the cast, and maintained superior flexibility even post-drying **(Figure 1)**. The average weight of the filmstrips was 41.7 ± 3.4 mg (Mean ± SD, n = 10). The weight of blank filmstrips averaged 24.8 ± 2.2 mg. As expected, filmstrip weight increased when the more dense allergen extract was incorporated.

The mean thickness of the strips was determined to be 78.0 ± 21.8 μm (n = 10). The thickness of the filmstrips tended to be more variable near the edges due to the effect of drying with the thickness at the center of the filmstrips measures 93.0 ± 13.4 μm. The films were cast using molds fabricated locally with limited selection in size, which constrained our ability to excise the desired dimensions while still avoiding edges. During cGMP manufacturing process, however, when much larger molds are employed, these variations in thickness would be significantly reduced. The typical thicknesses of blank filmstrips were 64.3 ± 9.8 μm.

### Foldability

Foldability of the filmstrips serves to ascertain the film's flexibility in order to sustain handling in the manufacturing process. Multiple strips with the highest protein extract content (25 μg/filmstrip) were tested, as flexibility decreases with increased extract percentage. The average number of folds the films can endure before cracking or complete failure was 11.0 ± 1.0 (n = 3).

The foldability of blank filmstrips was also tested. The blank filmstrips were much more flexible compared to their extract-containing counterparts, and the foldability for each of the three blank filmstrips exceeded 100 folds.

### Tensile strength

**Figure 2:**
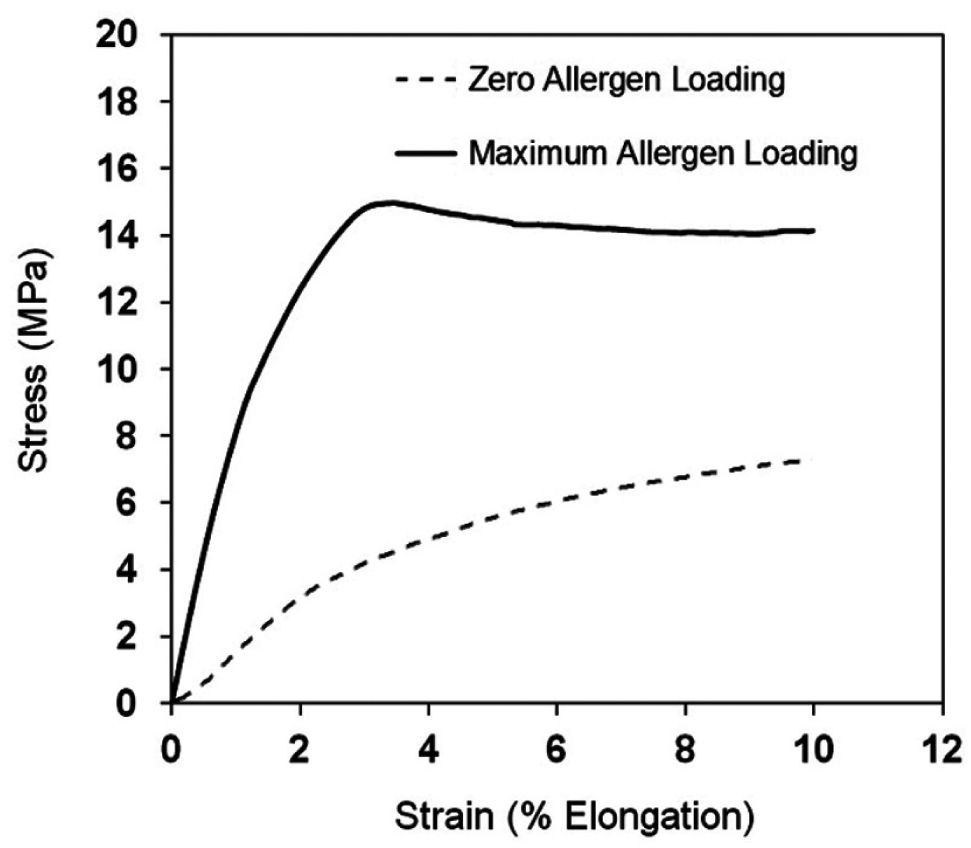
Typical Stress-Strain curve of SLIT thin films containing maximum (25 μg/filmstrip) aqueous cockroach protein extract and blank filmstrip without any allergen extract.

The ultimate tensile strength (UTS) is an important mechanical property for thin films. The UTS of the filmstrip with the highest protein loading (25 μg/filmstrip) was tested to be 15.0 ± 5.3 MPa (n = 9). Based on the behavior of the filmstrips we concluded that they possess sufficient physical integrity to allow easy processing and handling during manufacturing, transportation, and storage. The UTS of blank filmstrips were not reached at the end of 10% of elongation. Without the incorporation of the allergen extract, the blank filmstrips were much more ductile, and could be stretched to greater extents than those loaded with allergen. A typical stress-strain curve for a filmstrip with and without allergen extract is shown in **Figure 2**.

### Water content

Water content can directly influence the stability of the allergen in the filmstrips during storage. At the end of the film casting and drying process, the filmstrips were tested to contain on average 2.9 ± 0.9% of excess water in the formulation (n = 8).

### Surface pH

Surface pH indicates the degree of acidity of the filmstrip. The pH should be neutral or close to neutral as to not cause irritation to the oral mucosa during administration. The average surface pH of the filmstrips was 6.4 ± 0.3 (n = 3). The result is comparable to the pH of common drinking water, which is between 6.5 to 8.5, according to guidelines set out by the World Health Organization.

### *In vitro* disintegration test

The time it took for the film to visually break apart in water was recorded as the disintegration time. The mean disintegration time was 99.3 ± 16.6 seconds or 1.7 ± 0.3 minutes (n = 3). Based on previous experience, we defined a disintegration time of less than 5 minutes to be considered acceptable for this formulation. The disintegration time can be easily adjusted by tuning the composition of the film.

### *In vitro* dissolution test

**Figure 3:**
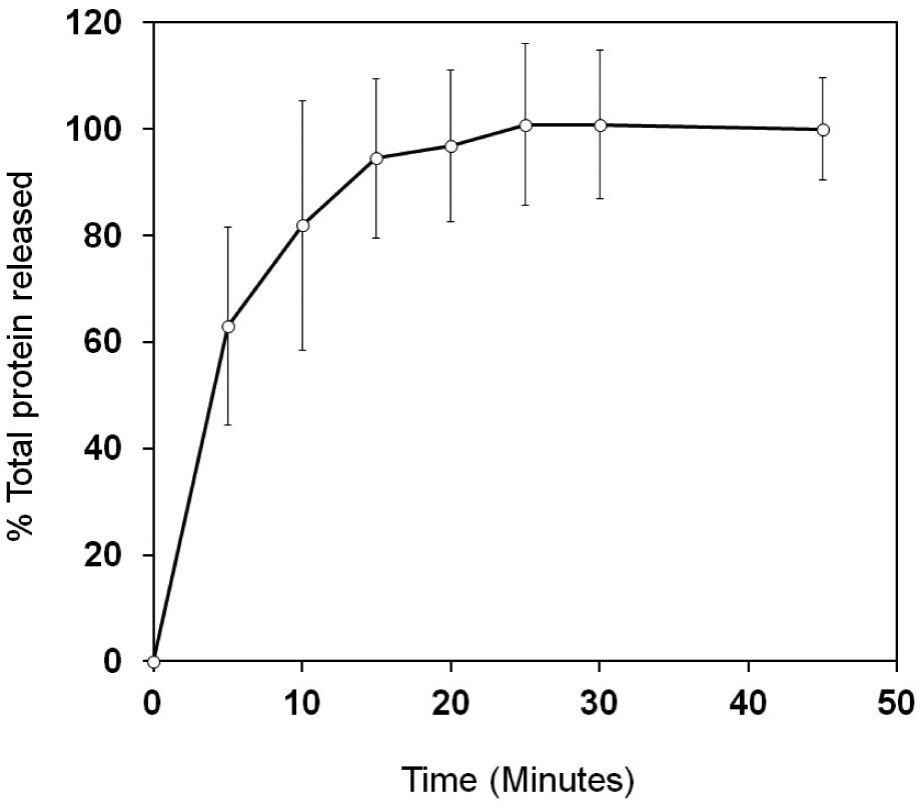
*In vitro* dissolution profile of sublingual immunotherapy filmstrips (n = 6). The results were quantitated against the standard curve for the total protein extract released.

The release profiles of the filmstrips are shown in **Figure 3**. In this formulation, 80% protein release was achieved in 15 ± 3 minutes, and total protein release was achieved within 25 minutes.

### Content Uniformity

Ten filmstrips with target Bla g 2 dose of 25 μg/filmstrip were selected and assayed by ELISA to determine the dosage uniformity. The relative standard deviation was 5.3%, which conforms to the acceptance criteria of less than or equal to 6.0%. The maximum allowed acceptance value for each individual preparation was no more than 15.0%. Based on these values, the tested strips were determined as having acceptable uniformity in their content of the Bla g 2 allergen.

### Stability

**Figure 4:**
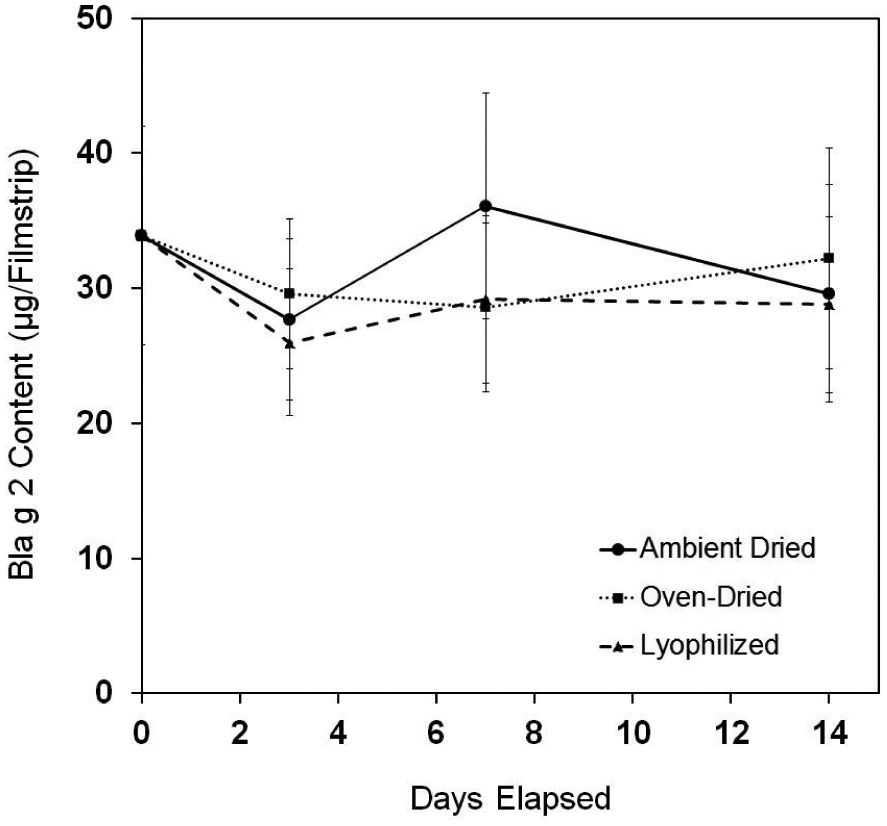
Decomposition profile of SLIT thin filmstrips under the accelerated stability test condition (40°C and 75% RH) over two weeks. The air-dried group of the filmstrips contained 5% excess water (●); oven-dried group contained 3% excess water (■), and lyophilized group of filmstrips contained no excess water (▲). Each data point is the average of two independent determinations of Bla g 2 allergen using ELISA.

The results of the accelerated stability study were summarized in **Figure 4**. The data compared the degradation of Bla g 2 protein in the thin film formulations with varying degrees of excess water content. The data clearly indicated that for the moisture contents studied the formulation was not sensitive to water content, and the filmstrips from all three groups remained stable at the end of the 2-week assessment. The filmstrips also did not exhibit significant changes in appearance, such as color, texture, or odor.

## CONCLUSION

Sublingual immunotherapy has rapidly gained popularity, since it is a noninvasive and efficacious treatment of common food and respiratory allergies [13]. In this study, the SLIT thin films could be formulated to contain up to 25 μg/filmstrip of Bla g 2 allergen, nearly 10 fold higher compared to the doses studied for liquid extracts. Visual examination showed that the fabricated filmstrips were smooth and lightly transparent with good flexibility. Physicochemical characterizations performed on the filmstrips revealed that the filmstrips were uniform in weight, thickness and allergen content, with allergen release profiles that met the preset requirements. The optimized filmstrip composition could bear stress well, and maintain physical integrity during processing such as bending and pulling.

The accelerated stability test for filmstrips processed to contain three different levels of excess mobile water showed no appreciable denaturation or degradation of the major allergen Bla g 2, regardless of the amount of excess water contained in the filmstrips. Therefore the manufacturing process of these strips can be done with fewer constraints but still be able to preserve the integrity of the final product. This greatly reduces the cost and manpower associated with manufacturing, and at the same time increase throughput of the final product.

In summary, the SLIT oral thin films reported here have high potential to be used to treat patients with hypersensitivity to cockroach. The films exhibit good mechanical strength, consistent potency and high stability. While only the cockroach allergen was studied in this report, this method may be used for SLIT in treating other types of food and inhalant allergens.

## Figures and Tables

**Table 1:** Composition of sublingual immunotherapy (SLIT) orally dissolving thin film.

Components	Formulation (w/v %)
Defatted cockroach extract	0 – 40
Methocel E15 LV Premium (10 w/v %)	50
Deionized water	49.8 – 9.8
Glycerin	0.15
Chocolate flavoring	0.5
